# Denoising of Fluorescence Image on the Surface of Quantum Dot/Nanoporous Silicon Biosensors

**DOI:** 10.3390/s22041366

**Published:** 2022-02-10

**Authors:** Yong Liu, Miao Sun, Zhenhong Jia, Jie Yang, Nikola K. Kasabov

**Affiliations:** 1 College of Information Science and Engineering, Xinjiang University, Urumqi 830046, China; leslie_lau@stu.xju.edu.cn; 2 Key Laboratory of Signal Detection and Processing, Xinjiang Uygur Autonomous Region, Xinjiang University, Urumqi 830046, China; 3 School of Physical Science and Technology, Xinjiang University, Urumqi 830046, China; sunmiao@stu.xju.edu.cn; 4 Institute of Image Processing and Pattern Recognition, Shanghai Jiao Tong University, Shanghai 200400, China; jieyang@sjtu.edu.cn; 5 School of Engineering, Computing and Mathematical Sciences, Auckland University of Technology, Auckland 1020, New Zealand; nkasabov@aut.ac.nz

**Keywords:** porous silicon, biosensor, quantum dots, image denoising

## Abstract

In the process of biological detection of porous silicon photonic crystals based on quantum dots, the concentration of target organisms can be indirectly measured via the change in the gray value of the fluorescence emitted from the quantum dots in the porous silicon pores before and after the biological reaction on the surface of the device. However, due to the disordered nanostructures in porous silicon and the roughness of the surface, the fluorescence images on the surface contain noise. This paper analyzes the type of noise and its influence on the gray value of fluorescent images. The change in the gray value caused by noise greatly reduces the detection sensitivity. To reduce the influence of noise on the gray value of quantum dot fluorescence images, this paper proposes a denoising method based on gray compression and nonlocal anisotropic diffusion filtering. We used the proposed method to denoise the quantum dot fluorescence image after DNA hybridization in a Bragg structure porous silicon device. The experimental results show that the sensitivity of digital image detection improved significantly after denoising.

## 1. Introduction

Porous silicon (PSi) is a new nanomaterial valued for its large specific surface area, good biocompatibility, and adjustable refractive index [1,2], and it has been widely used in the field of biological detection [3,4,5,6]. There are two main detection mechanisms of PSi optical sensors. The first is detecting refractive index changes caused by biological reactions [7,8,9,10]. The second type is the detection of fluorescence changes caused by biological reactions [11,12]. Both of these detection methods require spectroscopic instruments, which have a long detection time and high cost and cannot be used to detect sensor arrays. To reduce the detection cost, increase the detection speed, and realize the detection of porous silicon arrays, a method based on the gray value change of the transmitted light image was proposed [13]. This detection method can be used for PSi array parallel biological detection. In Reference [14], the author proposed a phagocytosis method to remove the speckle noise generated by the laser and porous silicon rough surface, which has been proven to improve the accuracy of image-based refractive index detection.

Reference [15] proposed a digital image gray value detection method based on quantum dot fluorescence labeling. The fluorescence image of the PSi surface was obtained with a digital microscope, and the target DNA concentration was detected by calculating the average gray value of the fluorescence image, thus achieving fast and low-cost biological concentration detection. Reference [15] proved that fast biological concentration detection can be realized by combining the image gray method and quantum dot labeling. To further reduce the detection limit of the quantum dot fluorescence image method, we take the suppression of noise generated by quantum dot fluorescence as the research subject.

In this paper, the distribution type of noise in the fluorescence image of quantum dots on the surface of porous silicon is determined by a convolution neural network. The influence of the noise on the gray value of the quantum dot image is analyzed. In the process of biological detection by digital image gray value method, the biological concentration information is mapped by detecting the gray difference before and after the biological reaction. Through our research, the noise after biological reaction will cause varying degrees of interference to the gray value of fluorescence image, resulting in low biological detection accuracy. As far as we know, there is no report on the denoising of quantum dot fluorescence images on the porous silicon surface. We propose a denoising method for quantum dot fluorescence images based on gray value compression and nonlocal anisotropic diffusion to solve this problem. The experimental results show that this method can recover the gray value affected by noise and significantly improve the accuracy of the biological detection method based on quantum dot labeling, which is of great significance to realize high-precision and low-cost biological detection. 

The content of this paper is organized as follows: Section 1 provides relevant background information. In Section 2, we analyze the noise of the quantum dot fluorescence image, and in Section 3, we introduce the proposed denoising algorithm in detail. In Section 4, we report our experimental results to demonstrate the excellent performance of the proposed method, and the conclusions are given in Section 5.

## 2. Theoretical Analysis

### 2.1. Detection of Noise Types in Fluorescence Emitted by Quantum Dots in Porous Silicon

In the process of biological detection using the quantum dot labeling method, quantum dots are randomly embedded into nanoholes with the size of 10–50 nm on the surface of porous silicon. Quantum dots fluoresce red when excited by blue light, and noise is generated along with the fluoresce signal. To explore the effect of fluorescent noise on the accuracy of biological detection and put forward the denoising strategy for fluorescent noise, we must first analyze the noise distribution type. In practical applications, there may be various noise types in the image, including Gaussian noise and salt and pepper noise, Poisson noise, speckle noise [16], or a mixture of these types of noise. Increasingly, research results show that the correct estimation of image noise is very important for image denoising, as it can reduce complexity and blindness and, moving forward, is essential for an effective targeted denoising algorithm. Studying the denoising model is the first work to put forward the denoising strategy [17,18]. With the continuous development of artificial intelligence in recent years, deep learning with high recognition accuracy has received increasing attention in image recognition. For example, the deep neural network implemented by the convolutional neural network has widely been used for image classification. Reference [19] proposed combining a principal component analysis filter and a CNN to recognize image noise. Although the data dimension reduction characteristics of principal component analysis are used, the training time of the whole network is still exceedingly long, and only four types of noise are tested. In [20], a residual learning framework RESNET is proposed to replan convolution. Generally, gradient disappearance occurs with the deepening of network layers, making parameters unable to be updated and training unable to converge to an optimal solution. RESNET avoids this situation through a short residual structure [21]. Experiments show that these residual networks are easier to optimize and greatly improve accuracy. To determine the type of noise in the fluorescence emitted by quantum dots in porous silicon, an image noise recognition algorithm based on RESNET was adopted in this paper. RESNET is currently a widely used CNN feature extraction network. Unlike other CNN networks, RESNET directly uses the parametric layer to learn the mapping between input and output and uses multiple parametric layers to learn the residual relationship between input and output. A short wire is added between multiple convolution layers to form a residual block, used as the basic unit of RESNET. Compared with other methods, this network model has the advantages of training time end, high precision, and short processing time.

To train the noise type recognition network, we established an image dataset with different noise levels and different noise types; the dataset used in this study includes gray values of 30, 40, 50, 60, 70, 80, 90, and 100. These eight monochromatic images with different gray values were added with 10 kinds of noise with different intensities—namely, Poisson noise, gamma noise, Rayleigh noise, exponential noise, uniform noise, Gaussian noise, salt and pepper noise, Gaussian and exponential mixed noise, Gaussian and salt and pepper mixed noise, gamma and salt, and pepper mixed noise—and labels were marked for corresponding noise types. A total of 2000 noisy images were constructed, and the dataset was composed of these images. Among them, 1700 noise maps were used as the training set for the RESNET network, and the remaining 300 were used as the test data. The test data were not included in the training set to verify the generalization performance of the network after training.

**Figure 1:**
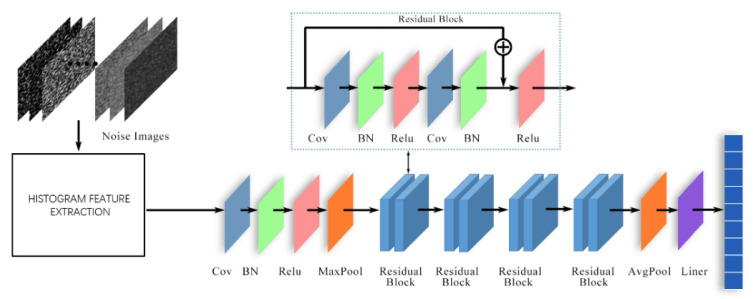
Residual network model for noise classification.

To classify noise, we used the statistical differences of various types of noise to extract the histogram features of the training image and used the RESNET network to further extract the features. The network included 17 convolution layers and a fully connected layer, which uses fast residual generation at its core. The fully connected layer comprised two convolution layers, two BN layers and two ReLU activation functions. The network composition diagram is shown in Figure 1. The learning rate is set to 0.001, the number of training iterations is set to 40, and the loss function is the cross-entropy function [22]. The SGD random gradient descent method was used in the optimization algorithm to improve the update speed of each round of parameters. This method can classify multiple noise types and has only 0.08 s running time and 99.8% accuracy. The output of the residual network was normalized by softmax:

(1) $$Softmax(Y_{i})=\frac{e^{Y_{i}}}{\sum_{k=1}^{K}e^{Y_{k}}}$$

where $Y_{i}$ is the output value of the ith node, and K = 10 is the number of output nodes, the number of categories. Through the softmax function, the output value of multiclassification can be transformed into a probability distribution in the range of [0, 1].

**Figure 2:**
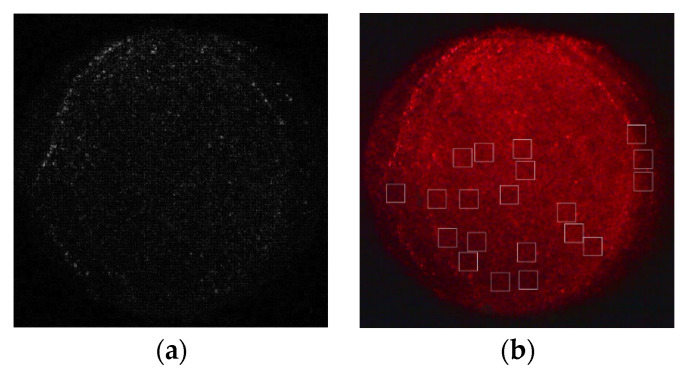
(**a**) Porous silicon image calculated by the eight-direction Laplacian; (**b**) a total of 20 homogeneous samples were selected.

The real sample used to judge the noise type was the fluorescence image of quantum dots on a porous silicon surface taken by an electron microscope in a biological detection experiment, as shown in Figure 2b. To improve the accuracy of the judgment, we used the homogeneous region of the fluorescence image as the experimental sample, so the experimental image data must be extracted and processed by high-pass filtering. Here, we used the Laplace operator, and the second derivative of the PSi fluorescence image was calculated with an eight direction Laplace operator; then, the high-frequency component was extracted, the dot product sum of the high-frequency graphs with a search window of size 20 × 20 was taken, the summation results were sorted, and 20 non-overlapping regions with the smallest result value were selected from all the search windows as the samples for noise type discrimination. The second derivative and sample selection calculation results are shown in Figure 2a,b, respectively.

To avoid the contingency of the experiment, we selected eight minimum gradient samples at different positions of the fluorescence images as the input of the residual neural network. The prediction results of the neural network are shown in Table 1. Through analysis of the experimental data, the classification results are very obvious. The ease of prediction of gamma noise is far greater than that of other types of distributed noise. We assumed that the noise type of the quantum dot PSi image is multiplicative gamma noise.

### 2.2. Influence of Gamma Multiplicative Noise on the Gray Value of Quantum Dot Fluorescence Images in PSi

Mathematically, image F polluted by multiplicative noise is the product of clean image U and noise N,

(2) F(i,j)=U(i,j)N(i,j)

where image F > 0 is the observed image, U > 0 is the original gray image, and N is the multiplicative noise following the gamma distribution with a mean value of 1 and variance of 1/L. Its probability density function is as follows:

(3) $$P(N)=\frac{1}{\Gamma (L)}L^{L}N^{L-1}e^{-LN}$$

where $\Gamma \left(L\right)=\int_{0}^{+\infty }t^{L-1}e^{-t}\mathrm{dt}$ is a gamma function and L >= 1. The mean value is E(N)=1. The variance is 1/L:

(4) $$\sigma_{N}^{2}=E[(N-E{\left(N\right)}^{2})]=\frac{1}{L}$$

**Figure 3:**
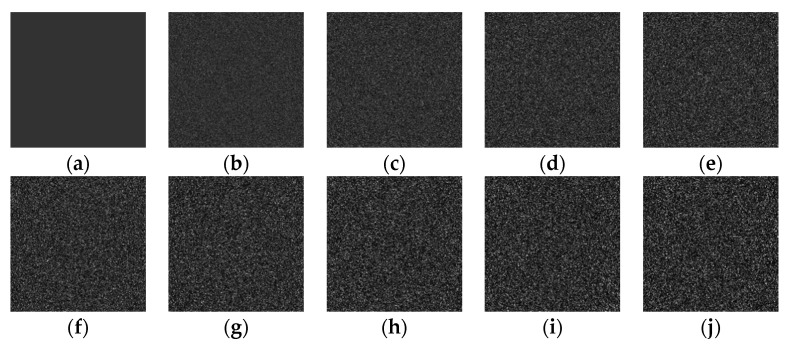
Gamma noise with different intensities is added to the gray image with a grayscale of 50: (**a**) original image, (**b**) var = 0.1, (**c**) var = 0.2, (**d**) var = 0.3, (**e**) var = 0.4, (**f**) var = 0.5, (**g**) var = 0.6, (**h**) var = 0.7, (**i**) var = 0.8, and (**j**) var = 0.9.

To verify the effect of multiplicative gamma noise on the gray level of the quantum dot fluorescence image in porous silicon, multiplicative gamma noise with intensities of 0.1, 0.2, 0.3, 0.4, 0.5, 0.6, 0.7, 0.8, and 0.9 was added to the gray level image with a gray level of 50. The gray level image with noise is shown in Figure 3.

**Figure 4:**
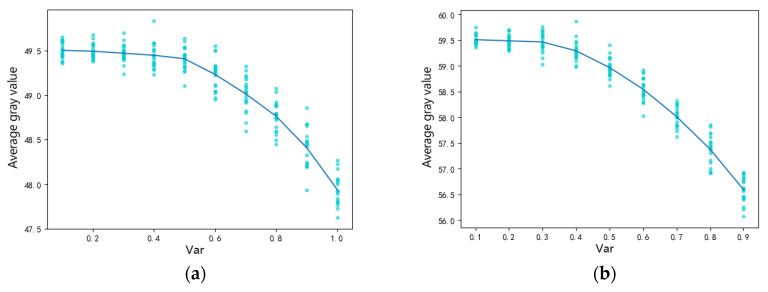
Influence of noise intensity between 0.1–0.9 on image gray value: (**a**) the influence of gamma noise on a gray value of 50 and (**b**) the influence of gamma noise on a gray value of 60.

To further explore the influence of multiplicative gamma noise on the overall average gray value, we added random gamma noise with a noise variance of 0.1–0.9 to gray images, calculated the average gray value, and repeated the 20 experiments. From Figure 4, we can see that the average gray value decreased with the increase in noise intensity, and the degree of decline gradually increased.

The above conclusions are significant for the biological detection of porous silicon sensors based on quantum dot fluorescence via imaging methods. The inner wall of nanoporous silicon with a Bragg structure used as a biosensor was functionalized in advance. First, the detected target biomolecule solution was fixed to the inner wall of the porous silicon via an adsorption mechanism. Then, the bio probe solution labeled with semiconductor quantum dots interacted with the porous silicon. The probe biomolecule was specifically bound to the target biomolecule, and the quantum dot was indirectly connected with the target biomolecule fixed to the hole wall. The probe molecules and quantum dots that did not specifically bind to the target biomolecule were washed out. When the porous silicon was irradiated by ultraviolet light, the quantum dots in the porous silicon were excited to produce red fluorescence. The Bragg structure can enhance the fluorescence emitted from the porous silicon surface. Using digital imaging equipment to obtain the fluorescence image of the porous silicon device surface, the average gray value of the image is directly proportional to the number of quantum dots in porous silicon, that is, directly proportional to the number of target biomolecules [15]. Accurate measurement of the average gray value of quantum dots on the surface of porous silicon devices is key in image detection technology. Gamma noise in fluorescence images reduces the average gray value and the sensitivity of biological detection, so it is very important to eliminate gamma noise in fluorescence images.

## 3. Proposed Denoising Algorithm

### 3.1. Related Research

In researching this form of noise removal, many remarkable algorithms have emerged, such as classical filters such as Kuan filter, Frost filter, Refined Lee filter, and guided filtering [23,24,25,26]. At the same time, many advanced denoising methods have emerged in recent years, such as the Perona–Malik model (PM) and speckle reducing anisotropic diffusion (SRAD) model [27,28]; block-matching and 3D filtering (BM3D) [29], which combines transform domain and spatial domain for denoising; probability-based nonlocal means filter (PNLM) [30], which combines nonlocal information, and to improve the denoising ability of nonlocal spatial information, an adaptive nonlocal spatial information denoising method based on the golden ratio is proposed [31]. Image restoration model based on total variation regularization [32], weighted maximum likelihood estimation [33], denoising method based on principal component analysis, and sparse representation [34,35,36,37] have also achieved good results in natural image denoising. With the development of deep learning, the convolutional neural network has also been applied to image denoising [38]. Based on the above experimental analysis, after adding multiplicative gamma noise to the gray image, the gray value of each pixel fluctuates near the original value under the influence of noise. The randomness of this fluctuation will lead to the change of the average gray value of the original image. The existing multiplicative noise removal methods can achieve a better subjective visual effect and better objective evaluation indices, such as peak signal-to-noise ratio (PSNR). However, for the special scene of quantum dot porous silicon biological detection fluorescence image denoising, the gray restoration effect of the existing algorithms is not ideal and even aggravates the gray deviation.

Given this, this paper proposes a method combining gray value compression (GVC) with nonlocal anisotropic diffusion based on cosine distance. GVC is used for image preprocessing to eliminate the local volatile singular pixels through limited compression processing and then use the anisotropic diffusion method to diffuse the noise. The traditional SRAD diffusion method takes the gradient operator and Laplacian operator of the image as the edge detection operator, so the calculated coefficient of variation is not robust. This paper proposes a new method to calculate the coefficient of variation, which can make full use of the nonlocal neighborhood structure similarity of each pixel. The new diffusion method is called nonlocal anisotropic diffusion based on cosine distance (CNLAD).

### 3.2. Proposed Denoising Algorithm

To eliminate the singular pixels in the image after adding noise and reduce the spatial fluctuation caused by multiplicative noise, we used gray value compression preprocessing measures for the image, so we had to find a compression reference standard. We used the noise image as the reference image for compression; the mean and Gaussian filters were ordinary smoothing filters. Considering that they are all based on local pixel information and are prone to blurring and diffusion at the edge, we used the nonlocal mean filter method to improve the accuracy of image pixel gray compression at the edge [39]. We set the search window size to 21 × 21 and the patch size to 7 × 7. The smoothing process is as follows:

(5) $${\tilde{I}}_{i}=\sum_{k\in I}w(i,k)I_{k}$$

where w(i,k) is the filtering weight of the kth pixel patch and the center pixel patch in the search window:

(6) $$w(i,k)=\frac{1}{Z(i)}e^{\left(-\frac{{\left\|I_{i}-I_{k}\right\|}^{2}}{h^{2}}\right)}$$

where $||I_{i}-I_{k}|{|}^{2}$ is the Euclidean distance between the center pixel patch and the kth patch, Z(i) is the normalization coefficient, and h is the smoothing parameter, which controls the attenuation degree of the Gaussian function.

The ratio of the original image input to the gray image filtered by nonlocal means is used as the basis of gray compression, which has a monotonically decreasing exponential relationship with the compression coefficient. We define $\gamma =e^{-\frac{I_{x,y}}{\tilde{I}}}$ as the compression coefficient; the larger the ratio is, the smaller the compression coefficient is, and the smaller the compression degree is. The smaller the ratio, the larger the compression coefficient and degree are. Singular pixels often correspond to larger compression coefficients.

**Figure 5:**
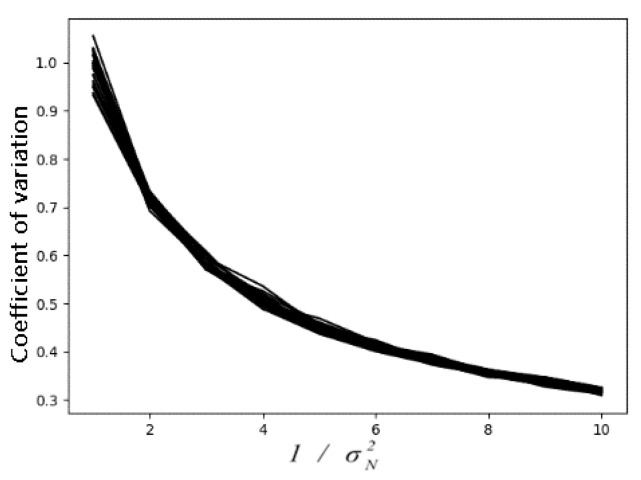
Relationship between the coefficient of variation and noise level.

The image after gray compression is expressed as

(7) $$I(x,y;k)=\left\{ \begin{array}{l} I(x,y;k-1)-\gamma \frac{I(x,y;k-1)}{10},\;I_{x,y}\geq \tilde{I}\\ I(x,y;k-1)+\gamma \frac{I(x,y;k-1)}{10},\;I_{x,y}<\tilde{I} \end{array}\right.$$

where $\tilde{I}$ is the image smoothed by nonlocal means, that is, the compressed reference image; $I\left(x,y;0\right)=I_{0}\left(x,y\right)$ is the original noisy image, and k is the number of compression iterations. Since the noise intensity cannot be directly obtained from the noisy image, we used the coefficient of variation in mapping for the noise variance. The coefficient of variation is a normalized measure of the dispersion degree of the probability distribution, reflecting the dispersion degree of the unit mean value. Through experimental adding noise to different gray images many times and with the increase in added noise variance, as shown in Figure 5, the noise variance has a good nonlinear relationship with the variation coefficient of the noisy image.

The coefficient of variation is defined as the ratio of the standard deviation and average value of the noisy image.

(8) $$\delta =\frac{\sqrt{\sigma^{2}}}{\mu }$$

**Figure 6:**
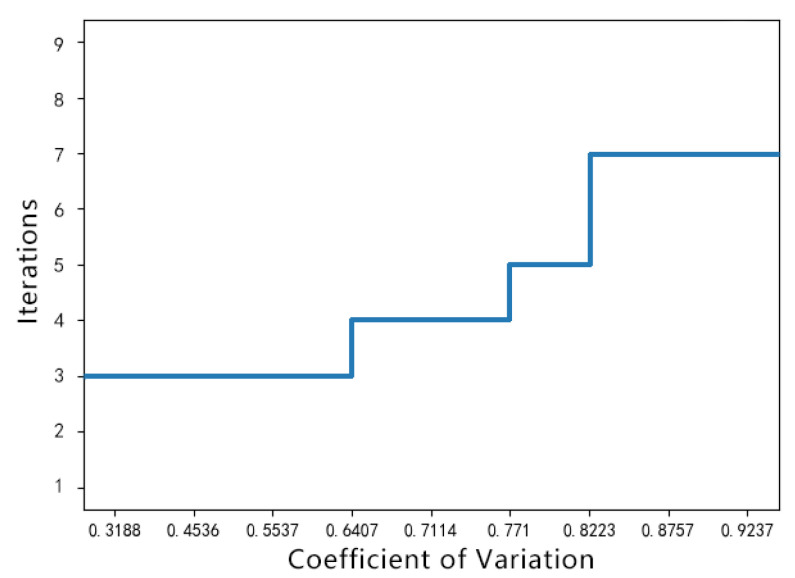
Relationship between number of iterations and the coefficient of variation.

In the actual experiment, to avoid the influence of heterogeneous regions on the estimation of the coefficient of variation, we used the Laplace operator to select P non-overlapping homogeneous regions, with a size of 20 × 20 in the image to estimate the coefficient of variation of the whole image, where P = 8, and Equation (10) is written as follows:

(9) $$\delta =\frac{1}{P}\sum_{i=1}^{P}\frac{\sqrt{\sigma_{i}^{2}}}{\mu_{i}}$$

where $\sigma_{i}^{2}$ and $\mu_{i}$ are the variance and mean value of the iTH homogeneous region of the noisy image. We used the coefficient of variation to determine the number of GVC iterations so that the peak of the gray histogram falls within the range of plus or minus 0.5 of the original gray value. We obtained the relationship between the number of GVC iterations and the variation coefficient through several compression experiments, as shown in Figure 6.

**Figure 7:**
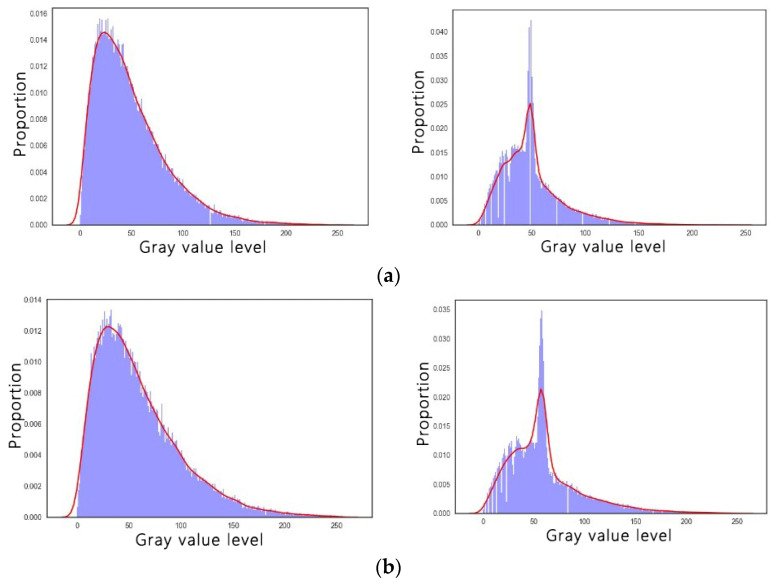
Histogram and probability density curve of the gray compressed image: (**a**) contrast histogram of the gray 50 noise image before and after compression; (**b**) contrast histogram of the gray 60 noise image before and after compression.

After gray value compression, the histogram features of the noisy image before and after gray compression were extracted and compared. The histogram is shown in Figure 7. It can be seen that the gray distribution in the histogram becomes relatively concentrated, which greatly reduces the fluctuation of the gray value in the noisy image, and the wave crest is close to the original gray value.

**Figure 8:**
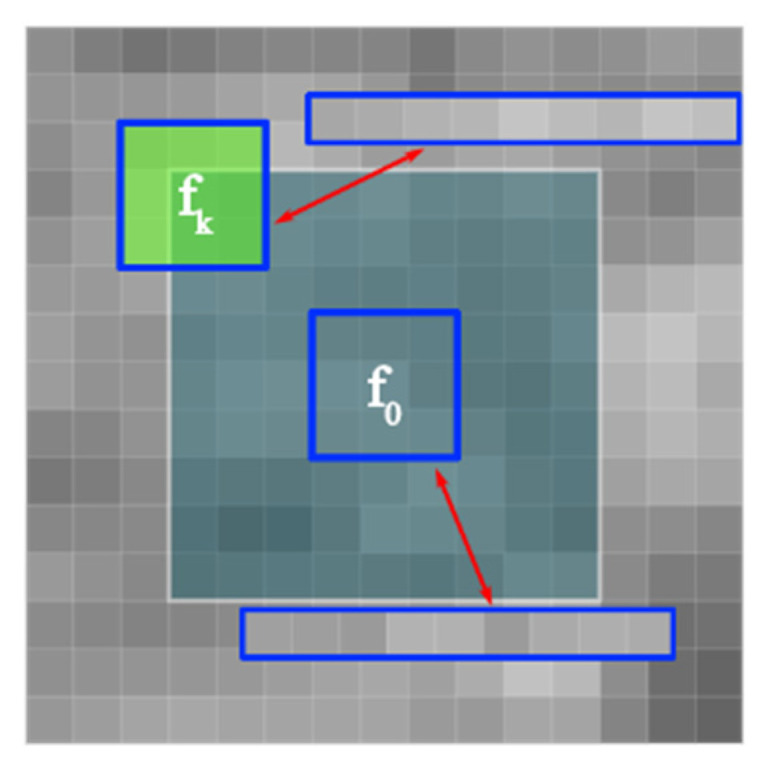
Nonlocal similar block matching diagram.

The main idea of the anisotropic diffusion model for denoising is to regard the image as a heat field and each pixel as a heat flow. We can determine whether to diffuse to the surroundings according to the relationship between the current pixel and the surrounding pixels. The anisotropic diffusion model of SRAD is as follows:

(10) $$\left\{ \begin{array}{l} \frac{\partial I(x,y;t)}{\partial t}=div(c(q)\times \nabla I(x,y;t))\\ I(x,y;0)=I_{0}(x,y)\\ {\frac{\partial I(x,y;t)}{\partial n}|}_{\partial \Omega }=0 \end{array}\right.$$

where div is the divergence operator, ∇ is the gradient operator, $I_{0}\left(x,y\right)$ represents the original image, I(x,y;t) represents the image at time t in the iterative process, ∂Ω represents the boundary of image region Ω, n represents the unit outer direction vector of the boundary, and c(q) represents the diffusion coefficient. The diffusion coefficient is expressed as

(11) $$c(q)=\frac{1}{1+\frac{q^{2}(x,y;t)-q_{0}^{2}(t)}{q_{0}^{2}(t)\left(1+q_{0}^{2}(t)\right)}}$$

where q(x,y;t) is the instantaneous variation coefficient, and $q_{0}\left(t\right)$ is the scaling function as follows:

(12) $$q(x,y;t)=\sqrt{\frac{{\left(\left|\nabla I\right|/I\right)}^{2}/2-({\left(\nabla^{2}I/I\right)}^{2})/16)}{{\left(1+(\nabla^{2}I/I)/4\right)}^{2}}}$$

(13) $$q_{0}(t)=\frac{\sigma (t)}{\mu (t)}$$

where σ(t) and μ(t) are the variance and mean value of the image at time t, respectively; the instantaneous variation coefficient of the SRAD diffusion model is calculated by using gradients of 0°, 90°, 180° and 270°. Although the local spatial gradient is effective for capturing local features, it only considers the range intensity similarity of two adjacent pixels. However, it does not consider the geometric structure similarity of two adjacent regions; it, therefore, lacks robustness [40]. Inspired by the nonlocal method, in this study, an effective adaptively weighted anisotropic diffusion model was constructed. The diffusion coefficient and instantaneous variation coefficient were determined by the cosine distance. A schematic diagram of the calculation of the local cosine similarity is shown in Figure 8.

The blue box $N_{f_{o}}$ represents the neighborhood of the pixel $f_{o}$, and the neighborhood size is 3 × 3. The white box $S_{f_{o}}$ is a square search box with 9 × 9 pixels centered on $f_{o}$. The cosine distance is used to measure the similarity of two regions. The cosine distance is also called cosine similarity. The cosine value of the angle between two vectors in vector space is used to measure the difference between two pixels. The expression is shown in Equation (14).

(14) $$d_{k}=\frac{\sum_{i}^{n\times n}f_{k}(i)\times f_{0}(i)}{\sqrt{\sum_{i}^{n\times n}f_{{}_{k}}^{2}(i)}\times \sqrt{\sum_{i}^{n\times n}f_{o}^{2}(i)}}$$

where f_o_ and f_k_ represent the central pixel block and the neighborhood pixel block, as shown in Figure 8, respectively; CNLAD uses the cosine distance between two blocks to measure the similarity between the neighborhood of the pixel in the search window and the neighborhood of the central pixel to achieve anisotropic diffusion edge detection. The moving step of the pixel patch in the search window is set to 1, n × n is the size of the pixel patch, and the mean value of the cosine distance is taken as the edge detection operator. At the same time, considering the influence of spatial distance on the similarity, the patch similarity of pixels close to the center pixel is given a larger distance weight, and the patch similarity of pixels larger than the center pixel block is given a smaller weight, that is, the cosine distance matrix is multiplied by a Gaussian kernel and then a weighted average.

(15) $$q_{x,y}=\frac{\sum_{k=1}^{S}d_{k}G_{k}}{\sum_{k=1}^{S}G_{k}}$$

$$G=\left[\begin{array}{ccccccc} 0.4 & 0.4 & 0.4 & 0.4 & 0.4 & 0.4 & 0.4\\ 0.4 & 0.6 & 0.6 & 0.6 & 0.6 & 0.6 & 0.4\\ 0.4 & 0.6 & 0.8 & 0.8 & 0.8 & 0.6 & 0.4\\ 0.4 & 0.6 & 0.8 & 1 & 0.8 & 0.6 & 0.4\\ 0.4 & 0.6 & 0.8 & 0.8 & 0.8 & 0.6 & 0.4\\ 0.4 & 0.6 & 0.6 & 0.6 & 0.6 & 0.6 & 0.4\\ 0.4 & 0.4 & 0.4 & 0.4 & 0.4 & 0.4 & 0.4 \end{array}\right]$$

where x and y represent the current pixel position; S represents the number of pixel patches in the search window; $d_{k}$ represents the cosine distance in the search window between the kth corresponding block and the target block. The diffusion threshold T can be obtained from the mean cosine distance of the whole image.

(16) $$T=\frac{1}{N\times N}\sum_{x=1}^{N}\sum_{y=1}^{N}q_{x,y}$$

The diffusion function is

(17) $$c(q)=\frac{1}{\sqrt{1+{\left(q(x,y;t)-T\right)}^{2}}}$$

where N × N is the size of the whole image, that is, the number of pixels participating in the cosine distance calculation; the diffusion coefficient formula of the anisotropic diffusion equation can be rewritten as

(18) $$c(q)=\frac{1}{\sqrt{1+{\left(q(x,y;t)-\frac{1}{N\times N}\sum_{x=1}^{N}\sum_{y=1}^{N}q_{x,y}\right)}^{2}}}$$

**Figure 9:**
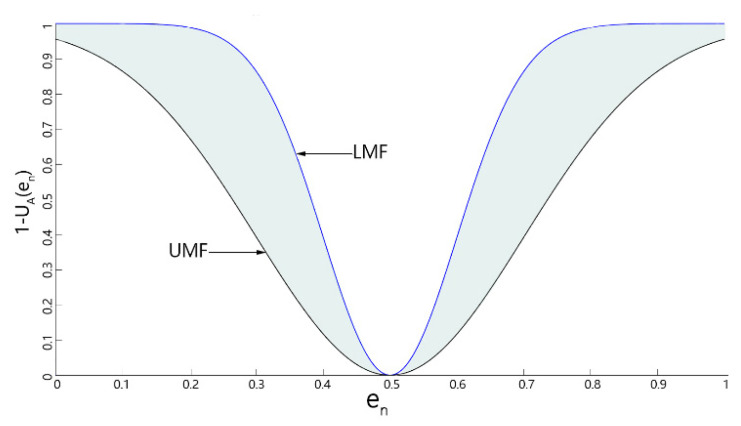
Membership function diagram.

For the preprocessed noisy image, the pixels affected by the noise will still fluctuate up and down in the original gray level. Considering the local pixel information, we proposed a method based on type-2 fuzzy, to judge the possibility that the pixels in the local neighborhood window are noise. A type-2 fuzzy system extends a type-1 fuzzy system, which is different from a type-1 fuzzy set. The membership degree of the type-2 fuzzy set itself is also described by the fuzzy set. Considering the input image I, the type-2 fuzzy set $\tilde{A}$ is characterized by the membership function $U_{\tilde{A}}\left(p_{i,j},U_{A}\right)$ and is defined as

(19) $$\tilde{A}=\{(x_{i,j},U_{A}),U_{\tilde{A}}(p_{i,j},U_{A})|\forall U_{A}\in J_{x_{i,j}}\subseteq \left[0,\;1\right]\}$$

where $U_{\tilde{A}}\left(p_{i,j},U_{A}\right)$ is the type-2 fuzzy membership function, and $J_{x_{i,j}}\subseteq \left[0,\;1\right]$ is the set of primary membership grades of $x_{i,j}\in I,\;\forall U_{A}\in J_{x_{i,j}}$. Reference [41] proposed mean of k-middle defined as follows:

(20) $$M_{K}(E)=\left\{ \begin{array}{l} \frac{1}{2K-1}\sum_{i=h-k+1}^{h+k-1}e_{i},\;if\;n\;is\;odd\;(n=2h-1)\\ \frac{1}{2K}\sum_{i=h-k+1}^{h+k}e_{i},\;if\;n\;is\;even\;(n=2h) \end{array}\right.$$

where $e_{i}$ is the ith order statistic of the n elements of E. For k = 1, the measure is the same as the classical median, and for k = h, the measure takes the form of the mean. Let us take pixel $x_{\mathrm{ij}}$ as the center; define the neighborhood window $W_{\mathrm{ij}}$ with window radius H = 1, and the membership degree of each element $e_{n}\left(n=\left(2H+1\right)*\left(2H+1\right)\right)\in W_{\mathrm{ij}}$ in the window forms a fuzzy set. We used Gaussian membership functions to calculate the membership degree as follows:

(21) $$U_{A}(e_{n})=e^{-{\left(e_{n}-\mu \right)}^{2}/2\delta^{2}}$$

where μ and $\delta^{2}$ are the mean and variance of Gaussian membership function. As shown in Figure 9, the upper bound UMF and lower bound LMF of type-2 membership function can be expressed by two primary Gaussian membership functions, which have the same mean value and different standard deviation, and the standard deviation is set as $\delta_{U}=0.2,\delta_{L}=0.1$ and $\mu =M_{2}\left(W_{\mathrm{ij}}\right)$; type-2 membership degree in a neighborhood pixel block can indicate the possibility that the pixel is noise to a certain extent. A large membership degree generally means that the pixel is less affected by noise. We took the membership degree as the reference of pixel diffusion degree, which can be expressed as

(22) $$U_{\tilde{A}}(e_{n})=\frac{UMF+LMF}{2}$$

**Figure 10:**
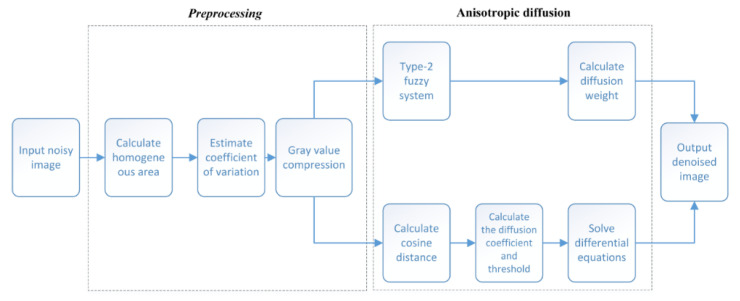
Algorithm flowchart.

The flowchart of our proposed denoising method is shown in Figure 10, and the diffusion process of pixels can be expressed as

(23) $$\begin{array}{l} I_{x,y}^{t+1}=I_{x,y}^{t}+\frac{\Delta t(1-U_{\tilde{A}}(e_{n}))}{4}(c_{x-1,y}^{t}(I_{x-1,y}^{t}-I_{x,y}^{t})+c_{x+1,y}^{t}(I_{x+1,y}^{t}-I_{x,y}^{t})+\\ c_{x,y-1}^{t}(I_{x,y-1}^{t}-I_{x,y}^{t})+c_{x,y+1}^{t}(I_{x,y+1}^{t}-I_{x,y}^{t})) \end{array}$$

## 4. Results and Discussion

### 4.1. Simulation Image Denoising

The algorithm flow chart is shown in Table 2, to verify the superiority of the proposed method in restoring the average gray value of the image. In this paper, six different algorithms were compared: BM3D [29], FFDNET [38], Refined Lee [25], PNLM [30], Adaptive TV [32] and PPB [33]. The results of the comparison algorithm are the optimal results after multiple parameter adjustments. In the FFDNET experiment, we did not use the original weight but retrained after adding gamma noise to the dataset. These six methods and the method proposed in this paper were used to process gray images with 50 gray levels containing multiplicative gamma noise with intensities of 0.1–0.9. The average gray level (AGL), root-mean-squared error (RMSE), and structural similarity (SSIM) were used to evaluate the denoising effect, and the RMSE indices are as follows:

(24) $$\mathrm{RMSE}=\sqrt{\frac{1}{M}\overset{M}{\underset{i=1}{\sum }}\left(y_{i}-{\hat{y}}_{i}\right)}$$

where $y_{i}$ is the ith pixel value, and M is the number of image pixels.

SSIM is composed of the luminance comparison (l), contrast comparison (c), and structure comparison (s) [42] as follows:

(25) $$\begin{array}{ll} SSIM= & l\left(x,\hat{x}\right)\times c\left(x,\hat{x}\right)\times s\left(x,\hat{x}\right)\\ & =\frac{2\mu_{x}\mu_{\hat{x}}+c_{1}}{\mu_{x}^{2}+\mu_{\hat{x}}^{2}+c_{1}}\times \frac{2\delta_{x\hat{x}}+c_{2}}{\delta_{x}^{2}+\delta_{\hat{x}}^{2}+c_{2}} \end{array}$$

where $\mu_{x}$, $\mu_{\hat{x}}$ is the mean value of pixel block x, $\hat{x}$; $\delta_{x}^{2}$, $\delta_{\hat{x}}^{2}$ is the variance of pixel block x, $\hat{x}$; $\delta_{x\hat{x}}$ is the covariance of x, $\hat{x}$; the values of C_1_ and C_2_ are constants to avoid instability when the denominator is close to 0.

**Figure 11:**
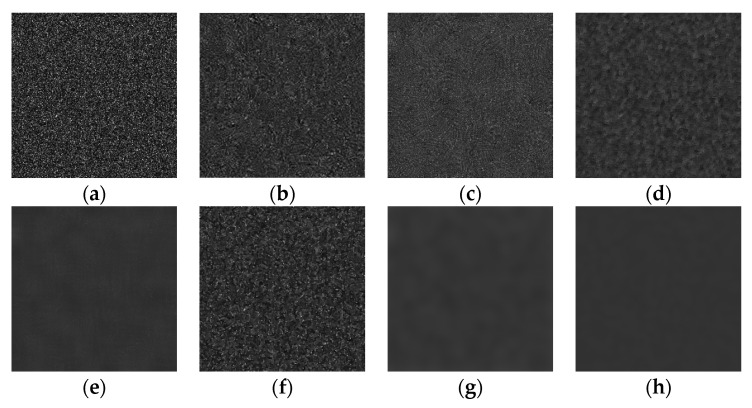
Comparison of different algorithms when the noise variance is 0.5: (**a**) unprocessed, (**b**) BM3D, (**c**) PPB, (**d**) Refined Lee, (**e**) PNLM, (**f**) Adaptive TV, (**g**) FFDNET, and (**h**) proposed.

Figure 11 shows the subjective denoising effect of other methods and our method when adding different intensities of multiplicative gamma noise to the gray image with a grayscale of 50. Compared with other algorithms, except BM3D and Adaptive TV, other algorithms have a certain degree of noise elimination effect in terms of the subjective visual effect.

**Figure 12:**
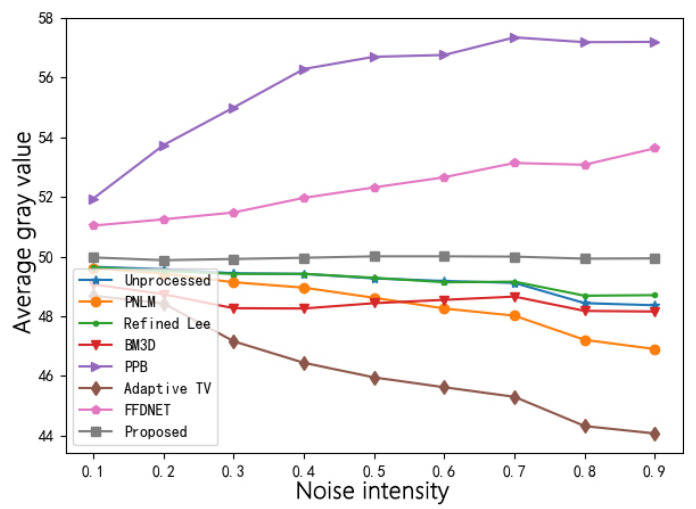
Comparison of gray restoration effect of various algorithms.

Figure 12 shows the changing trend of the image gray value after each algorithm’s processing with increasing multiplicative gamma noise variance, and Table 3 shows the AGL, RMSE and SSIM of the denoised image. The gray value presents an upward trend after the other six denoising methods, except PPB filtering and FFDNET, and the other five denoising methods further reduce the average gray value. Only the denoising algorithm proposed in this paper can remove the noise effectively and ensure the average gray value of the denoised image is close to the average gray value of the original image. Additionally, using the proposed method produces the results with the smallest RMSE value, which plays an extremely important role in the accuracy of biological detection based on the image gray value method.

### 4.2. Detection Using a Quantum Dot/Porous Silicon Optical Biosensor Based on Digital Fluorescence Images

**Figure 13:**
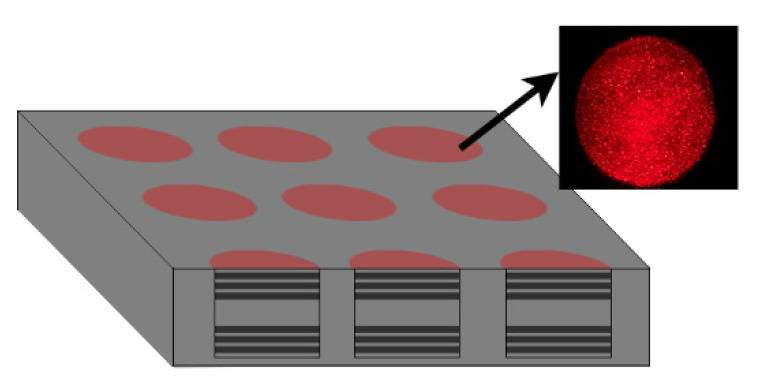
Bragg biosensor structure.

The preparation of a porous silicon Bragg biosensor, the coupling of semiconductor colloid QDs and probe DNA molecules, and the detection device of target DNA molecules for each imaging method were the same as those in [15]. The structure of the Bragg biosensor we prepared is shown in Figure 13.

**Figure 14:**
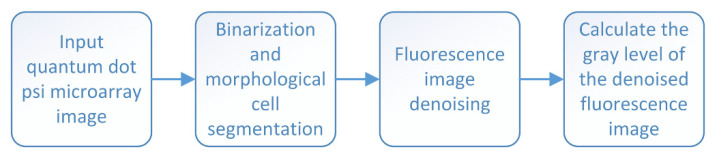
Flowchart of the real algorithm in the experiment.

We tested DNA samples with four concentrations of 0.1 nM, 0.25 nM, 0.5 nM, and 1 nM. To calculate the average gray value of the fluorescence image, first, its r-channel information was extracted and converted into the gray image. As shown in Figure 13, only the circular porous silicon unit area contained gray value information. We used the threshold binarization method to segment the porous silicon unit from the background and construct a rough binarization mask; the wrong segmented holes in the binary mask were etched and the edge of the array unit was smoothened, to separate the PSi array unit, which is convenient for denoising and calculating the gray level of each unit. The general flowchart is shown in Figure 14.

According to the theoretical analysis and the observation results of actual noise removal, it can be concluded that the algorithm proposed in this paper can effectively realize gray value recovery. Therefore, we applied this algorithm to real PSi fluorescence images.

**Figure 15:**
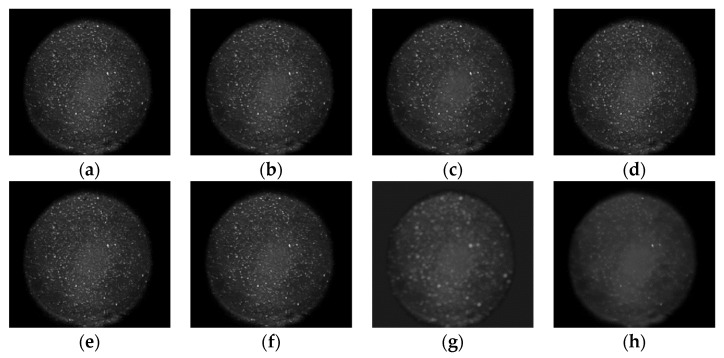
Comparison of the denoising effect of fluorescence image: (**a**) unprocessed, (**b**) BM3D, (**c**) PPB, (**d**) Refined Lee, (**e**) PNLM, (**f**) Adaptive TV, (**g**) FFDNET, and (**h**) proposed.

Figure 15a shows the fluorescence image of DNA detection with a concentration of 1 nM, and Figure 15b–h are the denoising results of BM3D, PPB, refined Lee filter, PNLM, adaptive TV, FFDNET, and our method, respectively. It can be seen from the denoised fluorescence image that the proposed method has the best denoising effect. For each group of samples, we took 10 images. After denoising with different denoising methods, we calculated the average gray difference of 10 images before and after the reaction and fitted these gray differences with the biological concentration.

The fitting formula and goodness of fit before treatment were as follows:

$$Y=9.21X+0.993\;R^{2}=0.988$$

After treatment, the fitting formula and goodness of fit were as follows:

$$Y=17.62X+0.837\;R^{2}=0.999$$

**Figure 16:**
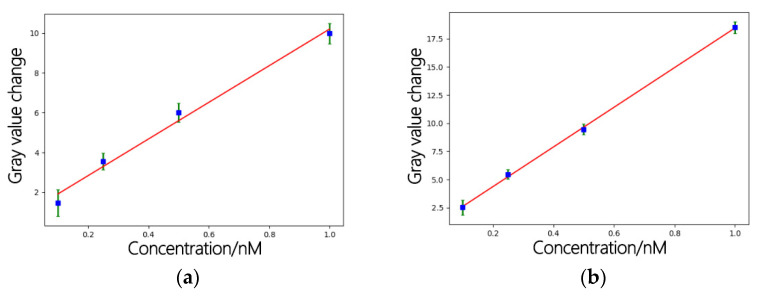
(**a**) The fitting curve between the gray value of the fluorescence image and the target molecular concentration before denoising; (**b**) the fitting curve between the gray value of the fluorescence image and the target molecular concentration after denoising.

Fluorescent images are different from other natural images in that they have rich texture information. In order to verify the effectiveness of the proposed denoising method for fluorescent images, we fit the curve between the average gray value of the denoised image and the biological concentration. Figure 16 shows the corresponding relationship between different biological concentrations and the gray value obtained after denoising algorithm processing. Compared with the fitting curve between the gray level of the fluorescence image before denoising and the target molecular concentration, the gray level of the processed fluorescence image has a good linear relationship with the target molecular concentration. We conducted experiments on four concentrations of DNA. For each concentration, we cut 10 silicon wafer samples under the same conditions and took the average value and standard deviation of the surface gray value of these 10 silicon wafers to obtain the error bar, as shown in Figure 16b; after the processing of this method, the fluctuation of noise on gray value is reduced, as is the error bar, while the goodness of fit between gray value difference and biological concentration is improved.

The PSi has an undulating surface, leading to accidental errors in the experimental process and affecting detection accuracy. The 3σ rule was used to compute the experimental results [43,44]. The minimum discernible grey value was 3σ, where σ was the standard deviation of the greyscale of the same sample for 10 consecutive measurements before the biological reaction. The detection limit is 3σ divided by slope. In our experiment, the measured σ value was 0.27, and the detection limit of DNA biomolecules was reduced from 87.9 pM to 46.0 pM according to the linear equation. It can be seen from the above experiment that the proposed algorithm is very suitable for recovering the gray levels of images affected by multiplicative gamma noise. The proposed algorithm can effectively reduce the influence of gamma noise in quantum dot fluorescence images and improve the biological detection accuracy achieved based on the image method.

## 5. Conclusions

In this paper, a residual convolution neural network model was developed to analyze the noise types produced by the fluorescence of quantum dots on the surface of silicon, and the influence of this type of noise on the average gray value of the image was analyzed. In this paper, a noise removal algorithm was proposed based on gray value compression and anisotropic diffusion for this kind of noise. The algorithm can effectively remove noise and restore the gray value to its maximum through simulation experiments. We fabricated a silicon Bragg mirror using an electrochemical etching method. The quantum dots were coupled with probe DNA and then combined with the target DNA fixed on the inner wall of the silicon hole. The biological detection device was used to detect the samples after the reaction, and the fluorescence image of the quantum dots was obtained. The detection limit of the DNA biomolecule was reduced from 87.9 pM to 46.0 pM after the fluorescence image was segmented and denoised by the algorithm proposed in this paper. This algorithm effectively improves detection accuracy and realizes low-cost and convenient biological detection.

## Figures and Tables

**Table 1:** Prediction results for the types of noise.

	Exp	Gamma	Rayleigh	Gamma+sp	Gaussian	Gauss+exp	Gauss+sp	Uniform	Poisson	S&P
Sample1	1.055 × 10^−3^	**9.034 × 10^−1^**	7.662 × 10^−2^	1.719 × 10^−3^	2.361 × 10^−4^	1.742 × 10^−4^	1.094 × 10^−2^	1.104 × 10^−4^	2.809 × 10^−3^	2.904 × 10^−3^
Sample2	9.049 × 10^−5^	**9.672 × 10^−1^**	1.849 × 10^−2^	6.203 × 10^−5^	3.576 × 10^−4^	6.414 × 10^−6^	5.235 × 10^−5^	5.415 × 10^−5^	4.456 × 10^−4^	1.315 × 10^−2^
Sample3	2.786 × 10^−7^	**9.992 × 10^−1^**	6.837 × 10^−4^	1.183 × 10^−6^	3.118 × 10^−6^	6.845 × 10^−8^	6.332 × 10^−8^	4.855 × 10^−7^	6.203 × 10^−6^	1.567 × 10^−5^
Sample4	2.791 × 10^−6^	**9.985 × 10^−1^**	1.226 × 10^−3^	1.968 × 10^−5^	2.983 × 10^−5^	4.828 × 10^−7^	1.149 × 10^−6^	2.719 × 10^−6^	3.363 × 10^−5^	1.288 × 10^−4^
Sample5	8.112 × 10^−6^	**7.762 × 10^−1^**	2.234 × 10^−1^	1.128 × 10^−6^	1.568 × 10^−5^	4.972 × 10^−8^	1.063 × 10^−6^	3.119 × 10^−5^	5.099 × 10^−5^	1.534 × 10^−4^
Sample6	4.535 × 10^−7^	**9.942 × 10^−1^**	5.722 × 10^−3^	4.006 × 10^−6^	1.786 × 10^−5^	4.157 × 10^−8^	6.264 × 10^−6^	4.195 × 10^−6^	7.960 × 10^−6^	2.220 × 10^−5^
Sample7	2.187 × 10^−5^	**9.891 × 10^−1^**	8.575 × 10^−3^	1.897 × 10^−5^	1.893 × 10^−3^	8.717 × 10^−7^	4.295 × 10^−6^	3.578 × 10^−4^	1.955 × 10^−5^	4.806 × 10^−6^
Sample8	3.763 × 10^−5^	**9.793 × 10^−1^**	1.170 × 10^−2^	7.331 × 10^−5^	3.871 × 10^−5^	6.360 × 10^−7^	1.562 × 10^−3^	6.296 × 10^−7^	3.483 × 10^−4^	6.830 × 10^−3^

**Table 2:** Algorithm flow.

Algorithm Flow
Step 1: The Laplace operator is used to obtain the mean region of the PSi image, and M blocks with the smallest gradient sum are used to calculate their coefficient of variation and take the mean value to estimate the coefficient of variation of the whole image, to determine the gray compression iteration number. Step 2: The input quantum dot PSi image is filtered by nonlocal means. The gray compression coefficient of each pixel is obtained by dividing the original image and the filtered image. The image is preprocessed using the GVC method. Step 3: Determine the search window with each pixel as the center, calculate the cosine distance between each pixel neighborhood and the center pixel neighborhood in the search window, determine the diffusion threshold T of anisotropic diffusion and the diffusion coefficient of each pixel, and solve the differential equation. Assess the probability that a pixel is a noise by the type-2 membership function and remove the noise with the anisotropic diffusion algorithm.

**Table 3:** Data statistics of nine gray pictures.

Var	Index	Unprocessed	PNLM	Refined Lee	BM3D	PPB	ADAPTIVE TV	FFDNET	Proposed
0.1	AGL	49.65	49.61	49.64	49.07	51.95	48.7	51.04	**49.97**
	RMSE	15.833	0.918	4.289	3.162	3.487	1.423	1.709	**0.279**
	SSIM	0.206	0.997	0.82	0.897	0.961	0.832	**0.998**	0.996
0.2	AGL	49.57	49.43	49.52	48.74	53.74	48.43	51.25	**49.88**
	RMSE	22.490	1.273	6.035	6.872	5.846	5.171	1.987	**0.506**
	SSIM	0.116	0.993	0.702	0.619	0.776	0.434	**0.997**	0.996
0.3	AGL	49.44	49.14	49.42	48.27	55.0	47.15	51.48	**49.92**
	RMSE	27.38	1.777	7.39	11.206	9.683	11.489	2.237	**0.589**
	SSIM	0.082	0.985	0.616	0.381	0.508	0.254	**0.996**	**0.996**
0.4	AGL	49.42	48.96	49.42	48.26	56.29	46.43	51.97	**49.96**
	RMSE	31.672	2.263	8.517	16.265	14.073	16.411	2.753	**0.723**
	SSIM	0.063	0.969	0.55	0.23	0.315	0.176	**0.995**	**0.995**
0.5	AGL	49.27	48.62	49.29	48.44	56.7	45.95	52.32	**50.01**
	RMSE	35.061	2.753	9.388	22.22	17.792	20.235	2.952	**0.737**
	SSIM	0.052	0.946	0.5	0.136	0.206	0.206	**0.995**	**0.995**
0.6	AGL	49.18	48.26	49.14	48.55	56.76	45.62	52.66	**50.01**
	RMSE	37.931	3.405	10.184	26.81	20.981	23.283	3.342	**0.796**
	SSIM	0.045	0.913	0.465	0.098	0.155	0.137	**0.994**	**0.994**
0.7	AGL	49.11	48.02	49.16	48.66	57.35	45.30	53.14	**50.00**
	RMSE	40.401	4.152	10.906	30.298	24.405	26.147	4.01	**0.954**
	SSIM	0.04	0.874	0.433	0.079	0.117	0.109	0.992	**0.994**
0.8	AGL	48.44	47.21	48.69	48.18	57.19	44.31	53.08	**49.93**
	RMSE	42.447	5.075	11.645	33.282	27.188	28.463	4.029	**0.998**
	SSIM	0.037	0.825	0.403	0.064	0.094	0.093	0.992	**0.994**
0.9	AGL	48.36	46.90	48.71	48.16	57.2	44.06	53.63	**49.94**
	RMSE	44.71	6.13	12.437	35.76	29.681	31.013	4.501	**1.096**
	SSIM	0.034	0.764	0.377	0.056	0.078	0.08	**0.989**	0.988

## Data Availability

The data presented in this study are available on request from the corresponding author. Data are not publicly available due to privacy considerations.
